# Modification of Poly(3-hydroxybutyrate-co-3-hydroxyvalerate)/Poly(lactic acid) Blends by In-Situ Reactive Melt Processing for Sustainable Packaging Application

**DOI:** 10.3390/ma19204406

**Published:** 2026-10-20

**Authors:** Maryam Abbasi, Erik R. Coats, Armando Gabriel McDonald

**Affiliations:** 1 Department of Forest, Rangeland and Fire Sciences, University of Idaho, Moscow, ID 83844, USA; 2 Department of Civil and Environmental Engineering, University of Idaho, Moscow, ID 83844, USA; ecoats@uidaho.edu

**Keywords:** poly(3-hydroxybutyrate-co-3-hydroxyvalerate), poly(lactic acid), reactive melt processing, chain extender, crosslinking, nucleating agent, rheology

## Abstract

**Highlights:**

- Additives (crosslinker, chain-extender, and talc) increased complex viscosity and improved the melt rheological behavior of PHBV/PLA blends.
- The commercial PHBV blend showed evidence consistent with limited insoluble network formation, while laboratory PHBV blend relied mainly on chain entanglement.
- Reactive melt processing reduced crystallinity in PHBV blends.

**Abstract:**

Poly(3-hydroxybutyrate-co-3-hydroxyvalerate) (PHBV) is a biopolyester produced by bacterial biosynthesis that has the potential to replace commodity plastics. However, poor melt strength and a narrow processing window limit its packaging application. In this work, commercial and pilot-scale PHBV is biosynthesized from fermented dairy manure using mixed microbial culture, extracted with dimethyl carbonate, and purified. Blends of the purified PHBV with poly(lactic acid) (PLA) were reactive melt-processed with triallyl isocyanurate (TAIC; 0.2–0.3 wt%), Joncryl (0.1–0.5 wt%), and talc (0.1 wt%), followed by injection molding. Rheological, thermal, and mechanical properties were studied and compared with modified commercial PHBV/PLA blends. The formulation containing 0.3% TAIC/0.3% Joncryl/0.1% talc showed the highest complex viscosity, indicating improved processability. Thermal analysis showed a broadened processing window for the laboratory PHBV/PLA blend but a reduction for the commercial blend. Molar mass analysis indicated considerable thermomechanical degradation, particularly in the laboratory blend. FTIR and gel fraction analysis were consistent with limited network formation in the commercial blend, whereas the laboratory blend showed no measurable gel fraction, with property improvements mainly attributed to enhanced chain entanglement. Differential scanning calorimetry showed reduced crystallinity in both blends. Mechanical testing showed reduced stiffness in both blends. Reactive melt processing enhanced the PHBV/PLA processability for sustainable packaging applications.

## 1. Introduction

Due to environmental pollution and sustainability issues associated with petroleum-based polymers, biodegradable biopolymers have attracted considerable attention as strong candidates to replace petroleum-based polymers in the packaging sector [1,2]. Since the packaging sector accounts for the largest plastic production market, with a share of around 50% of worldwide plastic waste, using biopolymers instead of petroleum-based polymers can make solving the plastic pollution problem much easier [3,4].

One of the leading candidates is poly(3-hydroxybutyrate-co-3-hydroxyvalerate) (PHBV), which is made from the monomers of 3-hydroxybutyrate (HB) and 3-hydroxyvalerate (HV). PHBV is a member of the poly(hydroxyalkanoate) (PHA) family, which is a bacterial polyester. PHAs are produced by bacterial biosynthesis when an excess carbon source is available and/or under limited nutritional conditions [5,6]. Current commercial production of PHBV relies on pure bacterial cultures and/or genetically modified strains under sterile conditions. It also requires refined carbon substrates such as glucose and fatty acids, which significantly increase production costs [7,8,9].

Recently, the use of mixed microbial cultures (MMCs) for PHBV production has gained attention as a more sustainable alternative, offering non-sterile conditions for PHBV production. The use of MMCs simplifies the production process and offers the potential for closed-loop production [10,11]. Utilizing renewable waste carbon substrates with MMCs can considerably reduce production costs—by more than 50% in some cases—depending on the substrate type, microbial community, and process parameters [10].

Properties such as biocompatibility, renewability, inherent biodegradability, and an excellent oxygen/vapor barrier make PHBV an attractive candidate for packaging applications and an excellent alternative to conventional petroleum-based plastics like polystyrene, polypropylene, and polyvinyl chloride [1,2,12]. However, inherent drawbacks such as poor melt elasticity, low degradation temperature, and the extreme brittleness of PHB and PHBV have limited its processability through various thermoforming processes, such as cast film extrusion, foaming, blown-film manufacturing, thermoforming, and fiber spinning [3,12]. A common approach to overcoming these limitations is to blend PHBV with other polymers to improve its mechanical and thermal properties.

To preserve the biodegradability of PHBV, it is usually blended with other biodegradable polymers, such as poly (lactic acid) (PLA) and poly(ε-caprolactone) (PCL) [13]. PLA is an aliphatic polyester that, due to its amorphous state, shows excellent thermoformability at normal processing conditions; thus, blending it with PHBV can be a suitable approach to enhancing PHBV’s thermoformability [14]. PCL has a low melting point (59–64 °C), high molar mass, and a semicrystalline structure. Furthermore, it is a hydrophobic, nontoxic, biocompatible, biodegradable polymer with a relatively low degradation rate. These properties have made PCL a valuable candidate in biomedical applications and a suitable modifier for improving the properties of other polymers such as PLA and PHBV [15,16,17,18,19].

However, the melt-blending of PHBV with PLA and/or PCL demonstrates immiscible behavior, which leads to poor interface adhesion and reduces the gas barrier of blended PHBV, when the PLA weight ratio is higher than 25% [12,20,21,22]. To address this problem, various additives have been used to improve the compatibility, crystalline structure, and processability of the blends [13]. Crosslinkers such as dicumyl peroxide (DCP), di(tert-butylperoxyisopropyl)-benzene (DB), and Luperox; chain extenders (CEs) such as a variety of Joncryl^®^ ADR series, triallyl isocyanurate (TAIC), and Graftalen^®^ MB series; and nucleating agents such as boron nitride (BN) [23], talc [23], calcium carbonate (CaCO_3_) [24,25], and silica [26,27] have been incorporated into PHBV or its blends to enhance its properties. These additives can improve the processability of the PHBV blend through various mechanisms, such as long-chain branching and polymer chain entanglement [2,21], reducing spherulite refinement, and improving the orderliness and density of crystalline regions [28]. Some CEs, such as Joncryl, can act as a compatibilizer as well. Compatibilizers help reduce the domain size of the dispersed phases and, therefore, enhance morphological stability [29].

An example of a PHBV/PLA blend with 0.25% Joncryl used as a CE improved 3D printability for fused filament fabrication and improved the mechanical properties [30]. The results showed a decreasing trend in crystallinity with PLA content. Furthermore, the optimized print composition was PHBV:PLA:Joncryl (40:60:0.25), which showed higher storage modulus and strength, resulting in stiffer and stronger parts when printed at 220 °C.

The application of talc as a nucleating agent in polymer blends can be found in numerous studies [31,32,33]. Chan et al. [31] enhanced the mechanical performance, water resistance, and structural integrity of PHBV/pine wood flour biocomposites by incorporating talc powder through a non-reactive strengthening approach. Talc provided space-filling morphology within the PHBV matrix, leading to improved composite stiffness. Further studies by Liu et al. [33] demonstrated that surface modification of talc powder significantly influenced the interfacial structure and mechanical properties of polypropylene/talc composites, indicating that tuning talc’s surface chemistry could optimize performance in polymer blends. Similarly, Hong et al. [32] applied in situ surface treatments to talc powder and showed improvements in the thermal and mechanical behavior of PLA/talc composites, supporting the potential for customized talc modifications in targeted polymer applications.

TAIC has been used as a crosslinking agent, in combination with a peroxide initiator in a PHBV/PCL blend through reactive extrusion [13]. The results showed improvement in interfacial compatibility, a significant enhancement in compatibility between PHBV and PCL as evidenced by a reduction in PCL particle size, and the disappearance of a distinct interfacial gap. At an optimal TAIC concentration of 0.99%, the compatibilized blend indicated an elongation at break 3.8 times greater than the unmodified PHBV/PCL blend and 7 times greater than neat PHBV. They attributed the improved toughness to the reduced PCL domain size, uniform PHBV crystallization, and formation of a PHBV/PCL copolymer structure, which makes it a strong candidate for use in sustainable packaging and related applications.

The aim of this study was to improve the rheological and thermal properties of pilot-scale produced and commercial PHBV with PLA by reactive blending. The modifiers used were TAIC as a compatibilizer; Joncryl ADR-4468 as chain extender; and talc powder as a nucleating agent. The modified blends were evaluated for improvements in rheological and thermal performance using injection molding to determine their potential for sustainable packaging applications.

## 2. Materials and Methods

### 2.1. Materials

**Figure 1:**
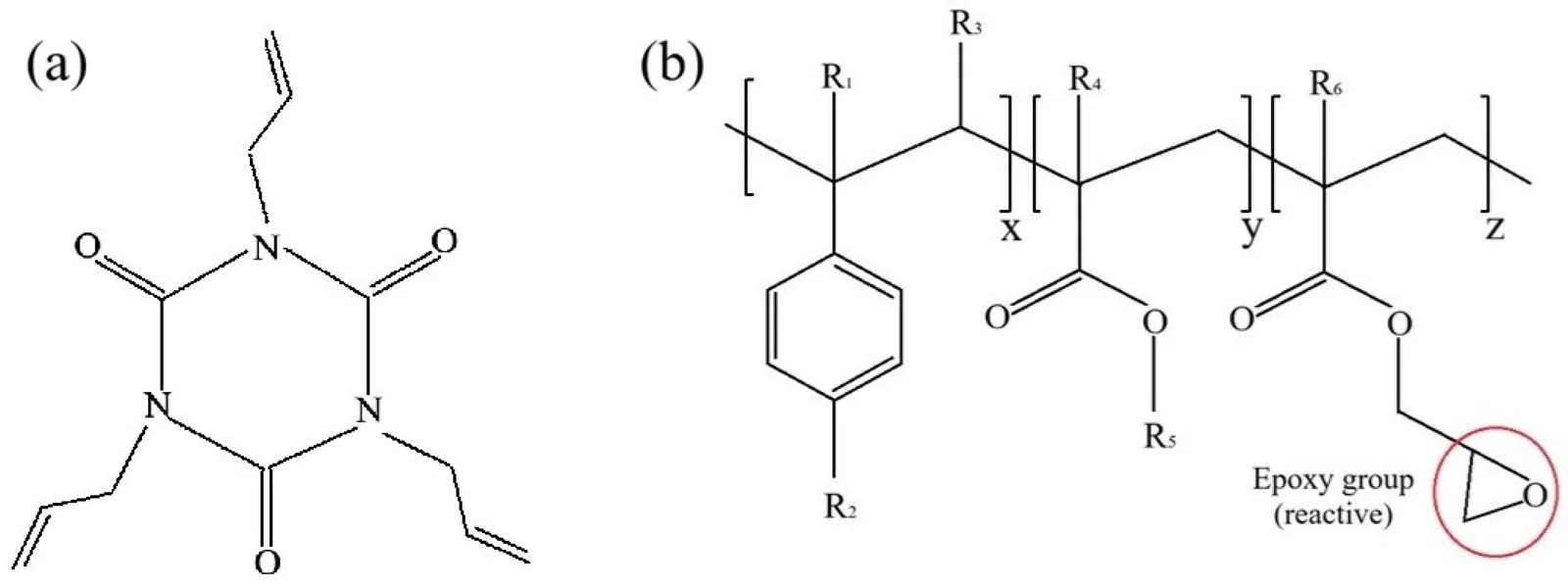
Chemical structures of (**a**) TAIC and (**b**) multifunctional epoxy chain extender Joncryl ADR^®^ 4468 (epoxy group highlighted by the red circle).

TAIC (Figure 1) was purchased from Tokyo Chemical Industry Co., LTD. (Tokyo, Japan). Multifunctional epoxide styrene acrylic oligomer, Joncryl ADR 4468^®^ CE (Figure 1), with a molar mass of 7250 g·mol^−1^ and epoxy equivalent weight 310 g·mol^−1^, was provided from BASF Co. (Florham Park, NJ, USA). Talc powder was provided from Luzenac (Paris, France). Commercial PHBV-8% HV pellets (C-PHBV) were purchased from Tianan Biopolymer (Ningbo City, Zhejiang Province, China). PLA was purchased from Purac Biochem (Lenexa, KS, USA).

### 2.2. Methods

PHBV containing biomass was produced in a pilot-scale system comprising (i) a sequencing batch dairy manure fermenter (760 L) to produce volatile fatty acids (VFAs), (ii) an enrichment bioreactor (680 L), which was fed VFAs to produce PHBV using an MMC, and (iii) a PHBV production reactor (450 L), which was also fed with VFAs in an MMC, as described by Guho et al. (2020) [11]. The laboratory PHBV (L-PHBV) was extracted from biomass using dimethyl carbonate in a modified Soxhlet system and purified by precipitation in cold methanol, as described in [34].

#### 2.2.1. Reactive Melt Processing

PHBV and PLA were dried in a vacuum oven at 60 °C overnight prior to processing. Since TAIC was in liquid form, it was dissolved in acetone (50 mL) to facilitate uniform dispersion of TAIC on the surface of pellets and to achieve the desired concentration (0.2–0.3 wt%). The TAIC solution was mixed with the PHBV/PLA blend, followed by evaporation of the acetone in a fume hood and then drying in a vacuum oven at 40 °C overnight. TAIC (0.2 and 0.3%), Joncryl (0.1, 0.3, 0.5 wt%), and talc powder (0.1 wt%) were blended with PLA/PHBV. The in situ reactive melt processing of PHBV blends (PHBV50/PLA50) with and without modifiers (2.0 g total weight) was performed using a Dynisco laboratory mixing extruder/molder (LMM) (Franklin, MA, USA) operating at 180 °C, with a mixing rate of 100 rpm for 3 min. The polymer melt was then injection-molded into microtensile dog-bone specimens in accordance with ASTM D1708 and discs (1.2 mm × 25 mm Ø) for rheology. For clarification, the sample coded as C-PHBV/TAIC 0.1/Joncryl 0.1/talc 0.1 was referred to as 0.1 wt% of Joncryl, 0.1 wt% of TAIC, and 0.1 wt% of talc powder incorporated into a commercial PHBV 50 wt%/PLA 50 wt% blend.

#### 2.2.2. Dynamic Rheology

The rheological properties of the injection-molded blends (25 mm Ø × 1.2 mm, two replicates) were evaluated using a dynamic rheometer (Bohlin CVO 100 NF, East Brunswick, NJ, USA) at 180 °C. A frequency sweep was conducted over the range of 0.01–100 Hz (0.0628–628 rad·s^−1^), with an applied strain of 0.5% to determine complex viscosity (η*) [13]. Data analysis was performed using Bohlin Rheology software (v6.51.0.3), and the results were subsequently fitted to a power law model using Microsoft Excel.

#### 2.2.3. Size Exclusion Chromatography

Size exclusion chromatography (SEC) multi-angle light scattering (MALS) analysis was used to determine the number (M_n_) and weight (M_w_), average molar mass, and polydispersity index (PDI) of the injection-molded samples (2 mg·mL^−1^, in duplicate). Separation was gained using a Jordi DVB linear mixed bed column (7.8 mm × 300 mm, Mansfield, MA, USA) with CHCl_3_ as the eluent (0.5 mL·min^−1^), coupled with a refractive index detector (Waters model 2414, Milford, MA, USA) and a multi-angle laser scattering (MALS) (PostNova Analytics PN3609, 658 nm, Landsberg, Germany) detector. A refractive index increment (dn/dc) value of 0.034 for PHB in CHCl_3_ was used [10,11,34]. Data analysis was performed with NovaSEC software (v1.5.0). The system was calibrated using a narrow polystyrene standard (Malvern, *M_w_* = 105,268 g·mol^−1^).

#### 2.2.4. FTIR Spectroscopy

The intermolecular interactions and crystallinity index (I_C=O_) of the vacuum-dried samples were analyzed by Fourier-transform infrared (FTIR) spectroscopy (ThermoNicolet iS5, Madison, WI, USA), using a ZnSe attenuated total reflection (ATR) accessory (ThermoNicolet iD5). Spectra were collected with 64 scans, obtained in duplicate, averaged, and baseline-corrected using Omnic software (v9.4, Madison, WI, USA). For quantitative analysis of the crystallinity index, the normalized spectra were curve-fitted with Igor Pro 9 software (WaveMetrics, Portland, OR, USA) [2,10,11,34,35]. The area (A) of each band obtained by curve fitting was integrated by the software. The crystallinity index (I_C=O_) was calculated as the ratio of the areas (A) under the carbonyl (C=O) absorption bands of PHBV in 1720 and 1740 cm^−1^ (I_C=O_ = A_1720_/A_1740_).

#### 2.2.5. Gel Fraction

A crosslinked polymer network is insoluble in organic solvents but swells into a gel. The gel fraction of the injection-molded samples was determined by refluxing in chloroform (CHCl_3_) for 24 h to remove the soluble “sol” fraction. The recovered gel fractions (coded as gel-PHBV) were vacuum-dried to a constant weight, and yields were calculated gravimetrically as follows:

Gel fraction (%) = (W_gel_/W_0_) × 100 (1)

where W_0_ is the dry weight of the crosslinked polymer, W_gel_ is the dry weight of the gel fraction [2].

#### 2.2.6. Thermal Analysis

The thermal properties of the blends were examined using differential scanning calorimetry (DSC, Q200, TA Instruments, New Castle, DE, USA). Samples (4–6 mg, in duplicate) were sealed in T_zero_ aluminum pans, equilibrated at −40 °C for 3 min, heated to 190 °C at 10 °C·min^−1^ (3 min hold), and subsequently cooled to −40 °C at −10 °C·min^−1^ (3 min hold), and the heating–cooling cycles were repeated. Data was analyzed with TA Universal Analysis software (v4.5A). The peak maximum of the exothermic and endothermic transitions in the second heating and cooling cycles was used to determine the glass transition temperature (T_g_), melting temperature (T_m_), crystallization temperature (T_c_), and degree of crystallinity (X_c_%). The X_c_ of each polymer in a blend was calculated using the following equation [35]:

X_c_% = ∆*H_m_*/(∆*H*_0_ × W_f_) × 100 (2)

where ∆*H_m_* is the heat of fusion of the sample, W_f_ is the weight fraction of polymer in the blend, and ∆*H*_0_ is the heat of fusion of a 100% crystalline polymer. The ∆*H*_0_ values used were 146.6 J·g^−1^ for PHBV [9] and 93.7 J·g^−1^ for PLA [2]. 

The thermal stability of neat polymers and blends was assessed by thermogravimetric analysis (TGA) using the Perkin–Elmer TGA-7 instrument (Shelton, CT, USA). Samples (3–6 mg, in duplicate) were heated from 30 °C to 800 °C at 20 °C·min^−1^ under nitrogen. Data was processed using Pyris software (v13.3).

#### 2.2.7. Mechanical Properties Testing

A tensile test was performed on the injection-molded microtensile dog-bone samples on a universal testing machine (Instron 5500R-1132, Norwood, MA, USA) equipped with a 5 kN load cell and a cross-head speed of 1.2 mm·min^−1^ in accordance with ASTM D1708 [13]. Strain was measured with a micro extensometer (model 3542, Epsilon Technology Corp., Jackson, WY, USA). Tensile data was analyzed with the Instron Bluehill v3.3 or v 4.4 software (Norwood, MA, USA). The results (at least 5 replicates) were statistically analyzed using the Tukey test (*p* < 0.05).

## 3. Results and Discussion

### 3.1. Rheology

To identify a suitable PHBV/PLA/modifier formulation for film processing, 13 compositions were prepared by varying the contents of TAIC (0.0, 0.2, and 0.3 wt%), Joncryl (0.0, 0.1, 0.3, and 0.5 wt%), and talc (0.0, 0.1 wt%) within the composition of C/L-PHBV50/50 PLA blends. These formulations were evaluated to determine their processing suitability. Since rheological behavior and thermal stability are critical indicators of processability, the results of these analyses were used to narrow the selection to the most promising compositions. The following discussion focuses on identifying the most promising formulation based primarily on its rheological response and thermal stability, and on comparing key findings, with particular emphasis on the influence of reactive modification and network formation on melt behavior.

**Figure 2:**
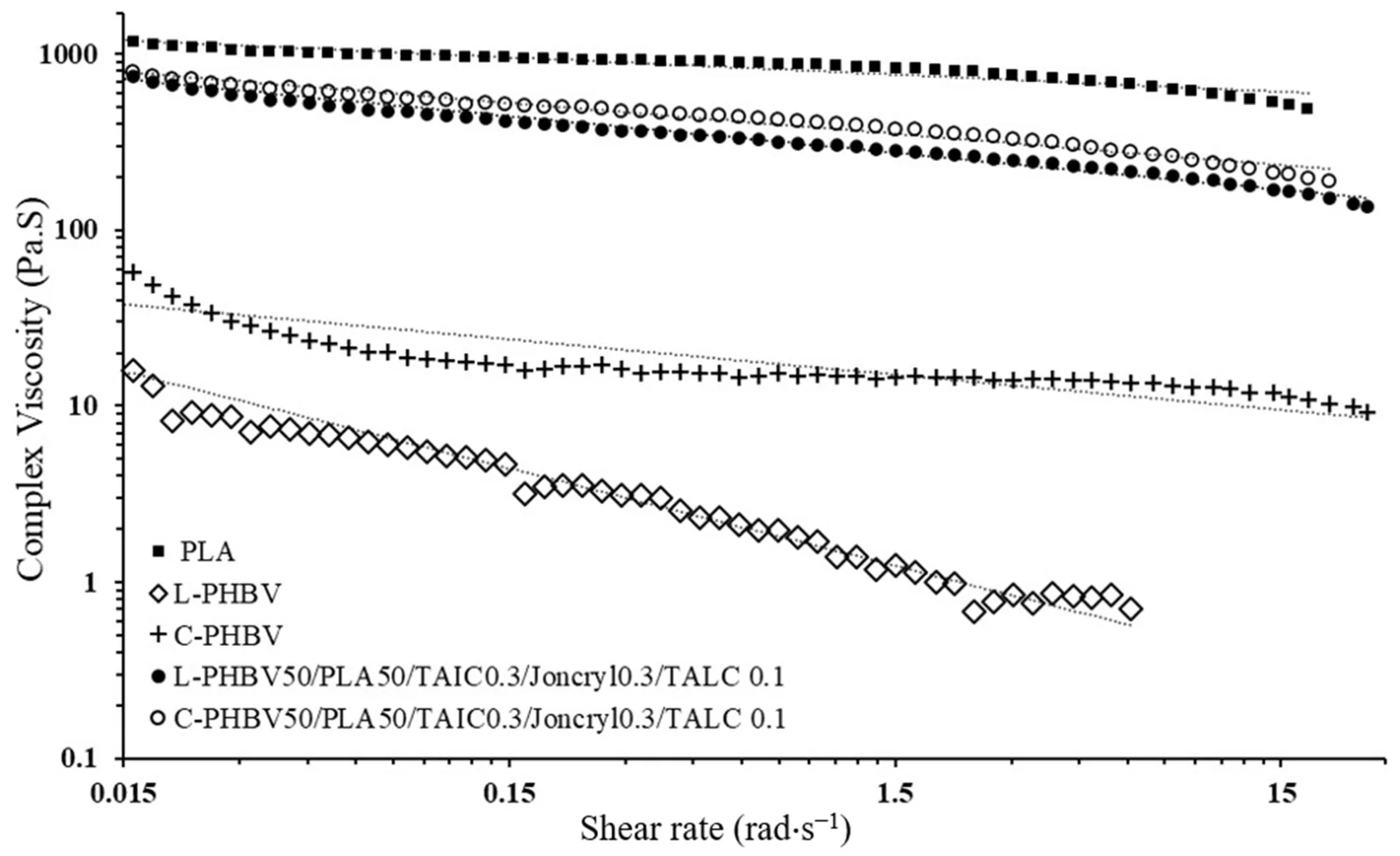
Complex viscosity (η*) versus shear rate (ω) plots for neat PHBVs, neat PLA, and optimized PHBV50-PLA50 compositions (180 °C), and power law-fitted models (dotted lines).

Rheological analysis provides an insight into polymer processability (e.g., injection molding) by characterizing the flow behavior [36]. Dynamic rheological measurements for the neat polymers and PHBV blends were conducted at 180 °C, and the complex viscosity (η*) was determined as a function of shear rate (ω), generating a flow curve (Figure 2). L-PHBV showed the lowest viscosity across the frequency range, followed by C-PHBV, whereas PLA displayed the highest viscosity. All modified formulations showed significantly improved viscosity relative to the neat PHBV samples, with flow curves positioned between those of neat PHBVs and PLA (Supplementary Material Figure S1). The flow curve of all compositions showed a decrease in η* with increasing ω, indicating shear thinning behavior characteristic of non-Newtonian, pseudo-plastic fluids [37]. At high shear rates, the anisotropic polymer chains with high molar mass will disentangle and align the shear direction, reducing intermolecular interactions and increasing free volume, which results in decreased viscosity [37,38]. The η* of L-PHBV samples decreased more rapidly with ω than the other formulations. This behavior can be attributed to its lower purity (92.9%), high HV content, and plasticizing effect of low molar mass PHBV present in the polymer, which increase chain mobility by enlarging the free volume between polymer chains [39]. Therefore, the polymer shows a sharper decrease in viscosity than the polymers without a plasticizer [40].

To quantitatively evaluate shear thinning behavior, the rheological data were fitted to the power law (Ostwald–de Waele) model, in which the η* is expressed as proportional to a power of the ω [41]. The relationship between steady shear and dynamic shear viscosity has been described using the Cox–Merz rule, which is applicable for PHBV, PLA, and their blends [34,41]. By exchanging steady shear terms with dynamic viscosity terms, a modified power law model was derived and expressed as:

|*η*^∗^ (*ω*)| = *K*(*ω*)*^n^*^−1^ (3)

**Figure 3:**
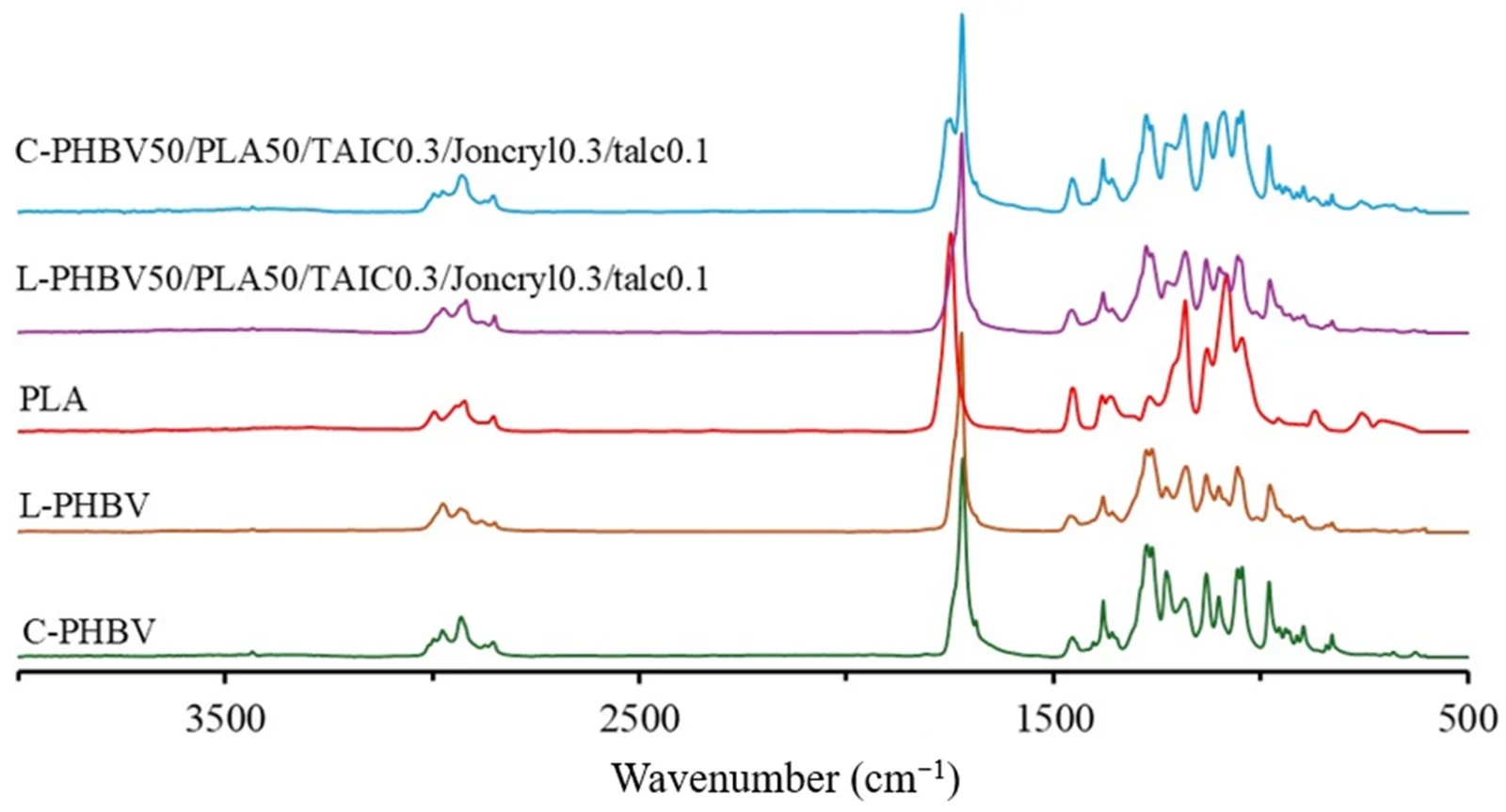
FTIR spectra of PLA, L-PHBV, C-PHBV, L PHBV50/PLA50/TAIC0.3/Joncryl0.3-/talc0.1, and C-PHBV50/PLA50/TAIC0.3/Joncryl0.3/talc0.1 samples before the gel content test.

The modified model has two parameters of *K* (consistency coefficient) and *n* (flow behavior or non-Newtonian index), which must be fitted to experimental data. These parameters were obtained from the double log plots of η* vs. ω (Figure 3). When *n* < 1, the material exhibits pseudo-plastic behavior; *n* = 1 indicates Newtonian behavior; and *n* > 1 corresponds to dilatant behavior [20,42,43]. However, one limitation of the power law model is that it considerably deviates from experimental data at the low and high shear regions and therefore should not be extended to these ranges [42,43]. The goodness of fit was evaluated using the model’s R^2^ value. All the PHBV compositions, except neat C-PHBV and L-PHBV, showed R^2^ ≥ 0.92, indicating good agreement with the model. C-PHBV and L-PHBV showed lower R^2^ values of 0.46 and 0.65, respectively. The fitted *K* and *n* parameters, along with η* at ω = 6.28 rad⋅s^−1^ for the various PHBV compositions, are summarized in Table 1.

The power law model fit the data well, with *K* values ranging from 1.55 to 338 Pa·s. The *n* values ranged from 0.448 to 0.903, confirming pseudo-plastic behavior for all formulations. For comparison, the η* of the PHBV compositions at ω = 6.28 rad⋅s^−1^ (1 Hz) was determined, with values ranging from 0.56 Pa·s to 665 Pa·s. The modified C-PHBV and L-PHBV formulations showed significantly higher η* than the corresponding neat polymers, with η* values of 273 Pa·s and 203 Pa·s, respectively. Among the evaluated formulations, the C-PHBV/PLA blend containing 0.3 wt% TAIC, 0.3 wt% Joncryl, and 0.1 wt% talc indicated the highest complex viscosity and so it was selected as the most promising formulation for the subsequent characterization. In contrast, L-PHBV showed the lowest η* (0.56 Pa·s), while C-PHBV showed η* of 11.3 Pa·s. An increase in melt viscosity observed in the modified PHBV blends can be partly attributed to blending with PLA, which displayed a significantly higher η* (665 Pa·s) compared with PHBV polymers.

The rheological behavior of the C-PHBV blend shows a complex competition between thermomechanical degradation and reactive modification. Although the Mw decreased, likely due to the high susceptibility of PHBV to random chain scission, the increase in the PDI value and the presence of a 3.73% gel fraction provide evidence consistent with chemical modification and limited network formation. Reactive melt processing with Joncryl and TAIC may have promoted chain branching and intermolecular network formation, which could restrict the molecular mobility of both C-PHBV and PLA. Simultaneously, crystalline domains of C-PHBV dispersed within the amorphous PLA phase act as physical crosslinking points, limiting chain mobility and promoting physical entanglements in the surrounding regions. Together, these chemical and physical interactions may increase the effective intermolecular interaction and overall entanglement density during melt blending. Consequently, despite the reduction in average chain length, the presence of branched clusters and crystalline obstacles results in longer relaxation times, leading to a significant increase in complex viscosity and enhanced melt rheological properties, particularly at low frequencies. This synergistic effect facilitates the formation of a pseudo-plastic network structure, in which the melt transitions from a liquid-like to a solid-like mechanical response as the frequency approaches zero [44,45,46].

In contrast, the modified L-PHBV blend showed a significant increase in η* without a corresponding increase in the PDI value or measurable gel formation. This suggests that reactive melt processing altered the melt structure without producing a measurable insoluble fraction. The Joncryl/TAIC system likely promoted limited chain extension and increased chain entanglement; however, the extent of these reactions was insufficient to generate a measurable gel fraction. The higher η* of the modified L-PHBV, compared with L-PHBV and comparable to that of modified C-PHBV, may be attributed to its higher HV content (28%). Higher HV content has been reported to enhance the miscibility of PHBV with PLA [47]. The improved miscibility promotes stronger chain entanglement between the PHBV and PLA phases during in situ reactive melt processing. Furthermore, the substantial reduction in crystallinity in modified L-PHBV (from 2.8% in L-PHBV to 0.08% after modification) indicates a predominantly amorphous structure, which may facilitate intermolecular interactions and chain entanglement between the L-PHBV and PLA phases [47,48]. Consequently, even in the absence of a measurable gel fraction, the changes in intermolecular interactions and melt structure in the optimized L-PHBV blend led to a viscosity increase that is nearly equivalent to that produced by gel formation in the optimized C-PHBV blend.

### 3.2. Molar Mass Determination

The L-PHBV had a purity of 92.9% and 28% HV molar ratio. The molar mass (M_w_ and M_n_) and molar mass distribution (PDI) of PLA, commercial PHBV, fermented PHBV, and their blends are given in Table 2. Changes in molar mass properties provide insight into the balance between chain scission, chain extension/branching, and possible network formation. The M_w_ and PDI values of C-PHBV, L-PHBV, and PLA were determined by SEC. C-PHBV had a M_w_ of 3.1 × 10^5^ g·mol^−1^ and a PDI value of 1.7, while L-PHBV M_w_ was 8.2 × 10^5^ g·mol^−1^ with a PDI value of 1.44. PLA was shown to have a M_w_ of 1.5 × 10^5^ g·mol^−1^ and a PDI value of 1.4.

As shown in Table 2, *M_w_* and *M_n_* of neat PLA showed no evidence of degradation as a result of injection molding under the processing conditions (*M_w_* = 1.8 ×10^5^ g·mol^−1^, and *M_n_* = 1.6 ×10^5^ g·mol^−1^). The negligible increase in both *M_w_* and *M_n_*, along with a slight PDI increase, indicates that the molar mass distribution remains narrow and stable. The PDI value for the PLA-pellet, before injection molding, was 1.14, and after injection molding it was 1.2. This shows that PLA maintains its chain integrity under the processing conditions used in this study (injection molding at 180 °C, 3 min).

The injection-molded neat commercial PHBV showed a slight reduction in *M_w_* with a constant *M_n_*, and a lower PDI value than the commercial PHBV pellet. The C-PHBV-pellet showed *M_w_* = 3.1 ± 0.0 × 10^5^ g·mol^−1^, *M_n_* = 2.6 ± 0.0 × 10^5^ g·mol^−1^, and PDI = 1.17 ± 0.0. For injection-molded C-PHBV, these numbers were *M_w_* = 2.9 ± 0.0 × 10^5^ g·mol^−1^, *M_n_* = 2.6 ± 0.0 × 10^5^ g·mol^−1^, and PDI = 1.10 ± 0.01. While the *M_n_* is constant, the reduced PDI value can suggest a minor degradation of higher molar mass chains and redistribution of chain lengths during processing. Overall, C-PHBV maintains relatively good stability under the applied processing conditions. The *M_w_* and *M_n_* of the blend of C-PHBV50/PLA50 were similar to the neat injection-molded PLA, with a slightly narrower PDI value, suggesting that blending alone did not produce considerable molar mass broadening. Compared to the binary blend of C-PHBV50/PLA50, the C-PHBV sample shows a mild reduction in *M_w_*, a significant reduction in *M_n_*, and a notable increase in PDI value. This behavior is consistent with the occurrence of competing simultaneous mechanisms during reactive melt processing, including TAIC-mediated chain branching and Joncryl-induced chain extension, which broadens molar mass distribution and PDI, together with thermal- and shear-induced random chain scission, which forms lower molar mass polymer chains, thus resulting in a reduced molecular weight [49,50]. Therefore, the results suggest that thermomechanical chain scission occurred concurrently with reactive modification and limited network formation. In PHBV and PLA, chain scission occurs in ester linkages. In PLA, this can occur through non-enzymatic random chain scission, and in PHBV, the dominant mechanism is random β-elimination involving six-membered ring transition, which results in the reduction in *M_w_* [49,50,51]. The reduction in *M_w_* indicates that thermally and shear-induced chain scission dominates over TAIC and Joncryl-induced chain extension and network formation [49,50].

L-PHBV showed a significant reduction in both *M_w_* and *M_n_* after injection molding. The neat fermented PHBV-pellet showed *M_w_* = 8.2 ± 0.3 × 10^5^ g·mol^−1^, *M_n_* = 7.0 ± 0.2 × 10^5^ g·mol^−1^, and PDI = 1.44 ± 0.38. These numbers dropped to *M_w_* = 3.1 ± 0.1 × 10^5^ g·mol^−1^, *M_n_* = 2.6 ± 0.1 × 10^5^ g·mol^−1^, and PDI = 1.20 ± 0.00. This substantial reduction in *M_w_* and *M_n_* after injection molding implies considerable thermal- and shear-induced chain scission over processing. Alongside the reduction in *M_w_* and *M_n_*, the reduction in the PDI value from 1.44 for the L-PHBV-pellet to 1.2 in the injection-molded L-PHBV indicates that degradation processes have eliminated high molar mass chains, shifted the average chain population to shorter chains, and so narrowed the chain distributions. Compared with PLA and the C-PHBV-pellet, the neat L-PHBV-pellet showed more sensitivity to melt processing under similar process conditions. The L-PHBV50/PLA50 blend showed molar mass values comparable to those of injection-molded neat L-PHBV and higher than those of PLA, with a narrow PDI value (PDI = 1.18). This indicates partial preservation of high molar mass chains when L-PHBV is processed in the presence of PLA. L-PHBV showed similar molar mass values to those of injection-molded L-PHBV, together with maintaining the PDI value at 1.2. The *M_w_* and *M_n_* in the L-PHBV blend were lower than the binary injection-molded blend of L-PHBV50/PLA50, *M_w_* (3.4 vs. 3.8 (×10^5^ g·mol^−1^)), and *M_n_* (2.7 vs. 3.2 (×10^5^ g·mol^−1^)), while the PDI value remained similar (1.20 vs. 1.18). The similarity of the molar mass values with the injection-molded L-PHBV suggests that, under the applied processing conditions, the thermal- and shear-induced chain scission mechanism dominated over chain extension and branching induced from TAIC and Joncryl [49,50], resulting in almost similar PDI values compared with the C-PHBV sample.

### 3.3. FTIR Spectral Analysis

FTIR spectroscopy was used to examine changes in characteristic functional groups and chemical environment in the studied polymeric compositions. Figure 3 shows the FTIR spectra of the optimized blends of L-PHBV and C-PHBV with PLA-C-PHBV50/PLA50/TAIC0.3/-Joncryl0.3/talc0.1 and L-PHBV50/PLA50/TAIC0.3/Joncryl0.3/talc0.1—prior to gel content testing. For comparison, the spectra of C-PHBV, L-PHBV, and PLA are also presented. These comparisons were used to examine possible copolymer formation induced by the additives during reactive melt processing. In particular, the FTIR analysis focused on identifying changes in band position, intensity, and the presence or absence of characteristic bands that could provide evidence of chemical modification in PLA/PHBV blends.

The FTIR spectrum of neat PLA showed the characteristic absorption bands associated with both crystalline and amorphous phases at 754 and 867 cm^−1^, respectively [52]. Additional PLA features included symmetrical C–O–C vibrations of the O–C=O group at 1043–1127 cm^−1^ [52,53] and symmetrical C–O–C stretching of the CH–O functional group at 1180 cm^−1^ [52,53,54]. The symmetric C–H bending vibration of methyl groups appeared between 1360 and 1381 cm^−1^ [53], while the symmetric –CH_3_ stretching of lactide was observed at 1452 cm^−1^ [53,55]. A major ester-related feature is the C=O stretching band at 1746 cm^−1^, corresponding to the amorphous carbonyl group of PLA [52,55,56]. The symmetric and asymmetric C–H stretching vibrations of CH_3_ groups appeared between 2852 and 2994 cm^−1^ [2,55,56]. In addition, the absorption region between 3501 and 3657 cm^−1^ is associated with O–H stretching vibrations [57].

The spectra of C/L-PHBV displayed several characteristic absorption bands corresponding to the PHBV backbone structure. The band at 910 cm^−1^ and the bands below 839 cm^−1^ [58,59] were assigned to C–H stretching and bending of CH_2_ groups. The band at 894 cm^−1^ corresponds to C–O stretching of C–O–C groups [60]. Peaks at 929, 953 [61], 977 cm^−1^ [35,60,62], and approximately 1043 cm^−1^ [63] are attributed to stretching and bending vibrations of C–C and C–H bonds. A weak absorption band at 938 cm^−1^ appeared only in commercial PHBV, which is associated with O–H bending vibrations [64]. The band observed at approximately 977 cm^−1^ corresponds to the C-C stretching vibration [35,62]. The C–O stretching vibrations occurred from 1054 to 1274 cm^−1^ [10,34,63,65,66,67,68]. The band at 1179 cm^−1^ corresponds to C–O–C stretching of amorphous PHBV [10,34,68]. Bands between 1357 and 1452 cm^−1^ were attributed to C–H stretching and bending vibrations of CH_3_ groups [61,62,63,65,66]. The strong carbonyl absorption peaks appeared at 1686 and 1717 cm^−1^ in C-PHBV, and 1720 cm^−1^ in L-PHBV, corresponding to C=O stretching of the ester group [10,34,35,36]. The symmetric and asymmetric stretching vibrations of –CH_3_ and –CH_2_ groups appeared in the 2852–2997 cm^−1^ range [10,62,65,69]. A narrow band at 3435 cm^−1^ observed in C-PHBV corresponded to O–H vibrations [59,60]. The FTIR spectrum of L-PHBV50/PLA50/TAIC0.3/Joncryl0.3/talc0.1 showed that the characteristic peaks of PLA, L-PHBV, and the additives remained largely unchanged compared to those of the neat components. This observation indicates that compared with the neat components, the characteristic peaks of PLA, L-PHBV, and the additives remained largely unchanged. Therefore, no clear FTIR evidence of significant chemical modification of the PLA and L-PHBV backbone was observed during reactive melt processing [13,69]. This finding is consistent with the absence of measurable gel fraction in the L-PHBV-based blend [13,69,70]. Although no chemical crosslinking was observed in the L-PHBV blend, minor shifts in the C–O and C–H-related absorption bands toward lower frequency, together with slight increases in band intensity, indicate changes in the local chemical environment or intermolecular interactions between PLA and L-PHBV, suggesting the presence of physical bonding interactions among PLA and L-PHBV [53]. Physical bonds may arise from hydrophobic, ionic, or protein interactions, stereo complex formation, hydrogen bonding, or crystallization effects [53]. These physical interactions can increase compatibility between the phases without producing crosslinking bonds detectable by FTIR spectroscopy.

**Figure 4:**
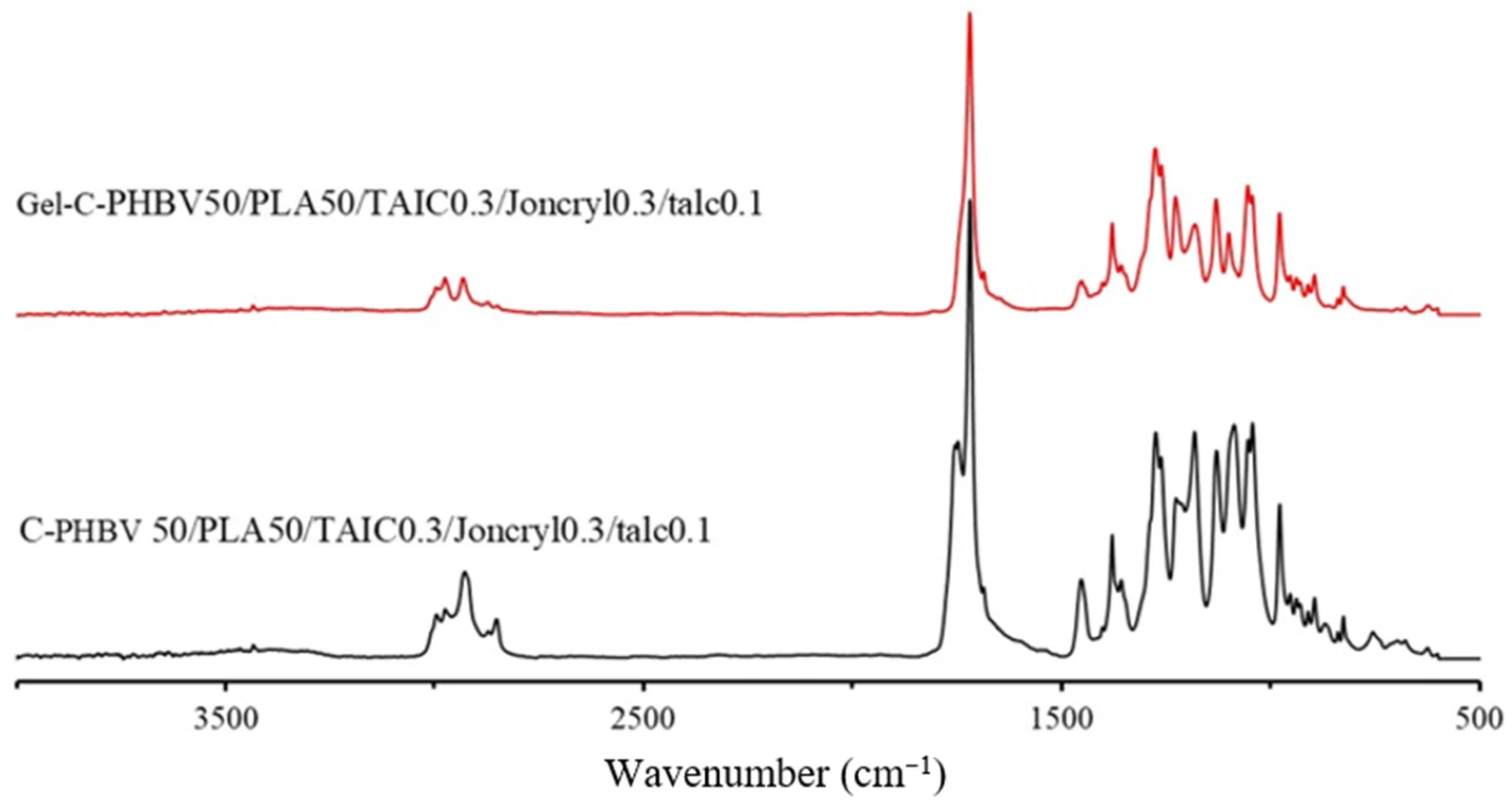
FTIR spectra of the C-PHBV50/PLA50/TAIC0.3/Joncryl0.3/talc0.1 sample before and after the gel content test.

**Figure 5:**
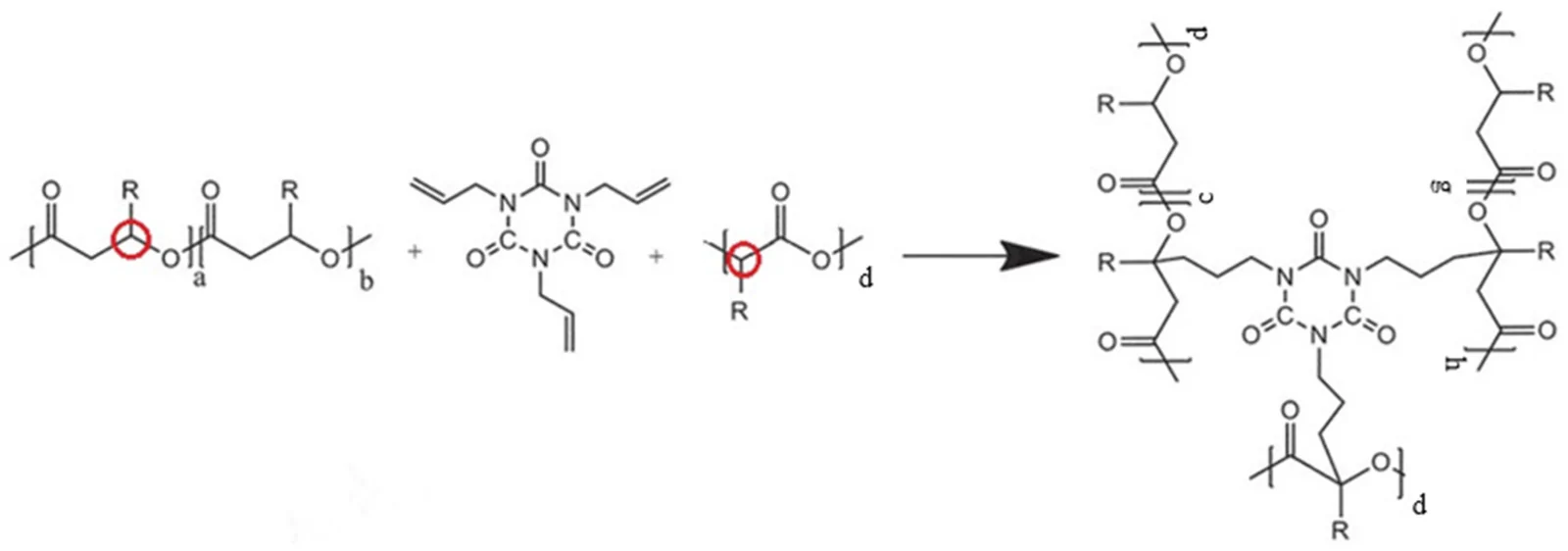
Possible reaction mechanism of TAIC with PHBV and PLA (R = CH_3_ or CH_2_CH_3_) and reaction site shown by a red circle (adopted and modified from [50]).

In contrast, the FTIR spectrum of C-PHBV50/PLA50/TAIC0.3/Joncryl0.3/talc0.1 showed some new bands beyond the characteristic bands of PLA, C-PHBV, and the additives, suggesting changes in the chemical environment and possible chemical modification during reactive melt processing. The gel fraction analysis provided additional evidence consistent with chemical modification. Compositions C-PHBV50/50 PLA–0.3 TAIC and C-PHBV50/50 PLA–0.3 Joncryl showed 0.4% ± 0.15 and 0.13% ± 0.18 gel fraction (two replicates, averaged), respectively, while the optimized composition C-PHBV50/PLA50/TAIC0.3/Joncryl0.3/talc0.1 showed a significantly higher gel fraction of 3.73% ± 0.71 (Figure 4). FTIR spectral analysis on the gel fraction (two replicates, averaged) showed the presence of the characteristic peaks of PLA, C-PHBV, and Joncryl and talc, indicating their incorporation into the isolated gel fraction. Several bands originally present in C-PHBV, namely 1805, 3390, 3515, 3634, and 3789 cm^−1^, were absent in the gel fraction spectrum. The band at 1805 cm^−1^ stands for C=O stretching related to thermal degradation products of PHBV produced during reactive melt processing [71,72]. Other mentioned bands are primarily associated with O–H stretching vibrations [70,73]. The disappearance of these bands in the gel fraction spectrum indicates that free hydroxyl groups were consumed during chemical reactions under reactive melt processing. Also, two new low-intensity bands appeared at 2750 cm^−1^ and 3195 cm^−1^, suggesting the formation of new chemical structures during reactive melt processing. The gel fraction spectrum further reveals that TAIC participated in the network formation. TAIC is a trifunctional crosslinking agent made from a triazine ring and three allyl groups [74,75,76]. It shows properties such as heat resistance, enhanced mechanical properties, and resistance to hydrolysis and weathering [76]. TIAC is widely used in thermoplastics and rubbers to increase thermal stability [74,75]. It typically crosslinks via the three reactive C=C double bonds of the allyl groups through a free-radical mechanism, where the double bonds open and form new covalent bonds with the polymer macroradicals, thereby bridging different polymer chains (Figure 5) [75]. It can be seen from the FTIR spectrum of the C-PHBV gel fraction that the band at 1644 cm^−1^, corresponding to C=C stretching of the allyl group of TAIC [54,75], disappeared, suggesting alteration of this unsaturated bond during reactive melt processing. The characteristic C=O symmetric stretching [54,75,77,78,79,80] and C-N stretching vibrations [77] of the isocyanurate ring appeared at 1681 cm^−1^ and 1448 cm^−1^, respectively, in the TAIC spectrum. In the gel fraction, these bands shifted slightly to 1686 and 1452 cm^−1^, suggesting the occurrence of a strong chemical interaction between C=O and PLA/PHBV [81], and thus indicating changes in the local chemical environment after insoluble network formation. Similarly, the 929 cm^−1^ band, associated with C=C bending vibration in TAIC [77] and C-H stretching in C-PHBV [82], showed a substantial reduction in intensity compared with the individual components (0.235 in TAIC and 0.131 in C-PHBV vs. 0.102 in the blend-gel fraction). This reduction indicates that the allyl double bonds participated in the chemical reactions during reactive processing. The band at 991 cm^−1^, corresponding to C=C bending vibration in TAIC [77], disappeared entirely in the gel fraction, further confirming the consumption of unsaturated double bonds during reactive processing. In TAIC, the bands at 3084 cm^−1^ correspond to C-H stretching of unsaturated C=C bonds [83,84]. This band disappeared in the gel fraction, indicating that the C-H bonds associated with double bonds were converted into saturated C-H single bonds during reactive melt processing.

**Figure 6:**
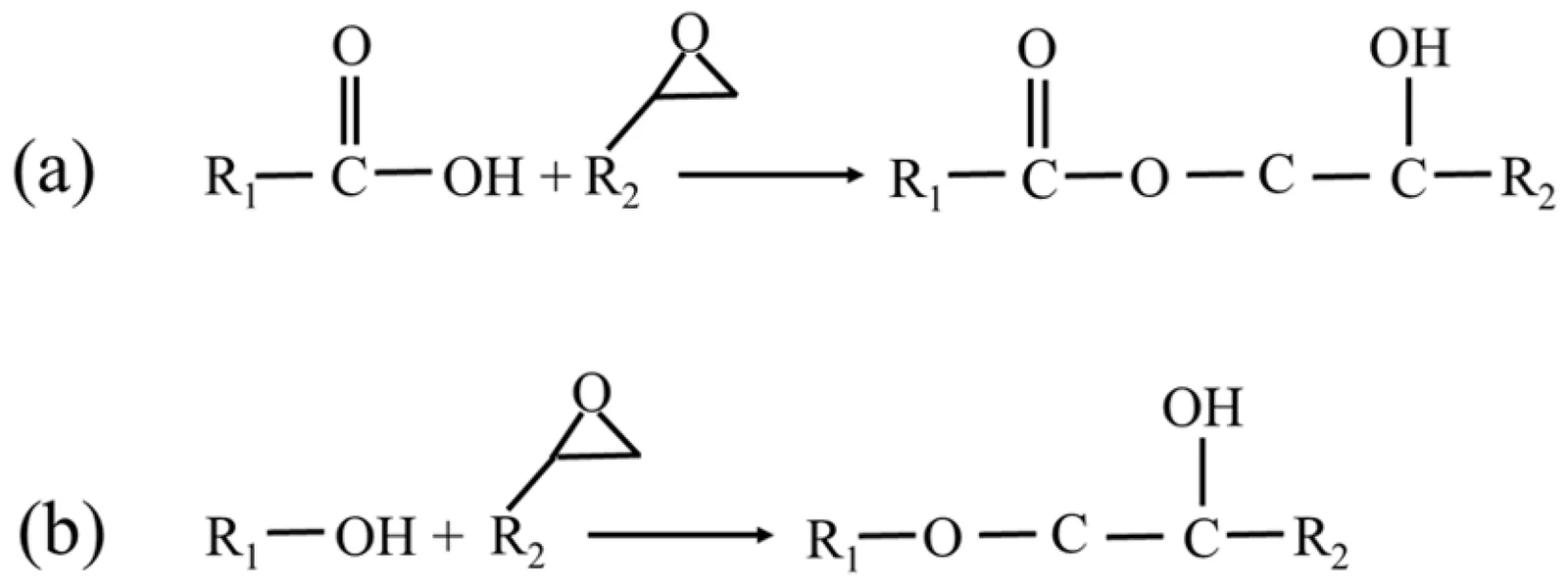
Reaction mechanism of a multifunctional epoxy chain extender with (**a**) the carboxyl end group and (**b**) the hydroxyl end group in PLA and PHBV (R_1_ = PLA or PHBV, R_2_ = Joncryl ADR type).

The FTIR spectral results show that Joncryl also contributed to structural modification of C-PHBV during the reactive melt processing. Joncryl is a styrene-acrylate copolymer containing multiple epoxy functional groups per chain, which are highly reactive toward carboxyl (-COOH) and hydroxyl (-OH) end groups of PLA and PHBV polymers (Figure 6). Through an epoxy ring-opening reaction, Joncryl can act as a chain extender, linking polymer chains and forming branched structures [32,85,86,87]. Comparison of the Joncryl spectrum with that of the gel fraction of C-PHBV shows that the epoxy-related bands at 844 and 907 cm^−1^ disappeared, indicating epoxy ring opening and formation of stable C–O ether or ester linkages [85,86,87]. The band at 1254 cm^−1^, corresponding to C–O stretching of the epoxy group [85,86,87], also disappeared or overlapped with neighboring peaks from PLA and C-PHBV (symmetric C–O–C stretching of crystalline PHBV [65,70,88] and C–O stretching of O–C=O in PLA) [87,89]. In the individual components, this region appeared at 1261 cm^−1^ in C-PHBV and 1266 cm^−1^ in PLA, while in the C-PHBV composition, all the three bands shifted to 1262 cm^−1^. The reduced intensity of this band relative to the combined intensities of the individual components further supports the participation of Joncryl epoxy groups in chain-extension reactions. Epoxy ring opening also generates new hydroxyl (–OH) groups within the polymer network. Consequently, the FTIR spectrum of the gel fraction showed increased intensity in the 3200–3600 cm^−1^ region, which is assigned to O–H stretching vibrations [90]. The new band at 3195 cm^−1^ likely reflects the formation of hydroxyl groups produced through reactions between the epoxy groups of Joncryl and the carboxyl end groups of PLA and PHBV. A second new band at 2750 cm^−1^ in the gel fraction spectrum corresponds to aldehyde C–H stretching and indicates that additional chemical transformations occurred during reactive melt processing. This band may originate from the conversion of –CH_2_ groups in C-PHBV and PLA [13] or from mild thermal degradation or chain scission processes during melt processing, which can generate new –COOH, –OH, and aldehyde end groups within the polymer chains [50,77,91,92]. These mechanisms collectively explain the appearance of the 2750 cm^−1^ absorption band and further support the occurrence of chemical reactions during reactive melt processing. Reactive modification and chain-extension reactions also produced slight changes in the carbonyl region of the spectra. The symmetric C=O stretching band of crystalline C-PHBV at 1717 cm^−1^ [35,47,60,61,65,88] shifted slightly to 1719 cm^−1^, accompanied by a small reduction in intensity. This shift reflects alterations in the chemical environment and hydrogen-bonding interactions within the more rigid insoluble network structure. Formation of a three-dimensional network restricts chain mobility, resulting in slight shifts and broadening of the C=O band.

### 3.4. Thermal Properties

**Figure 7:**
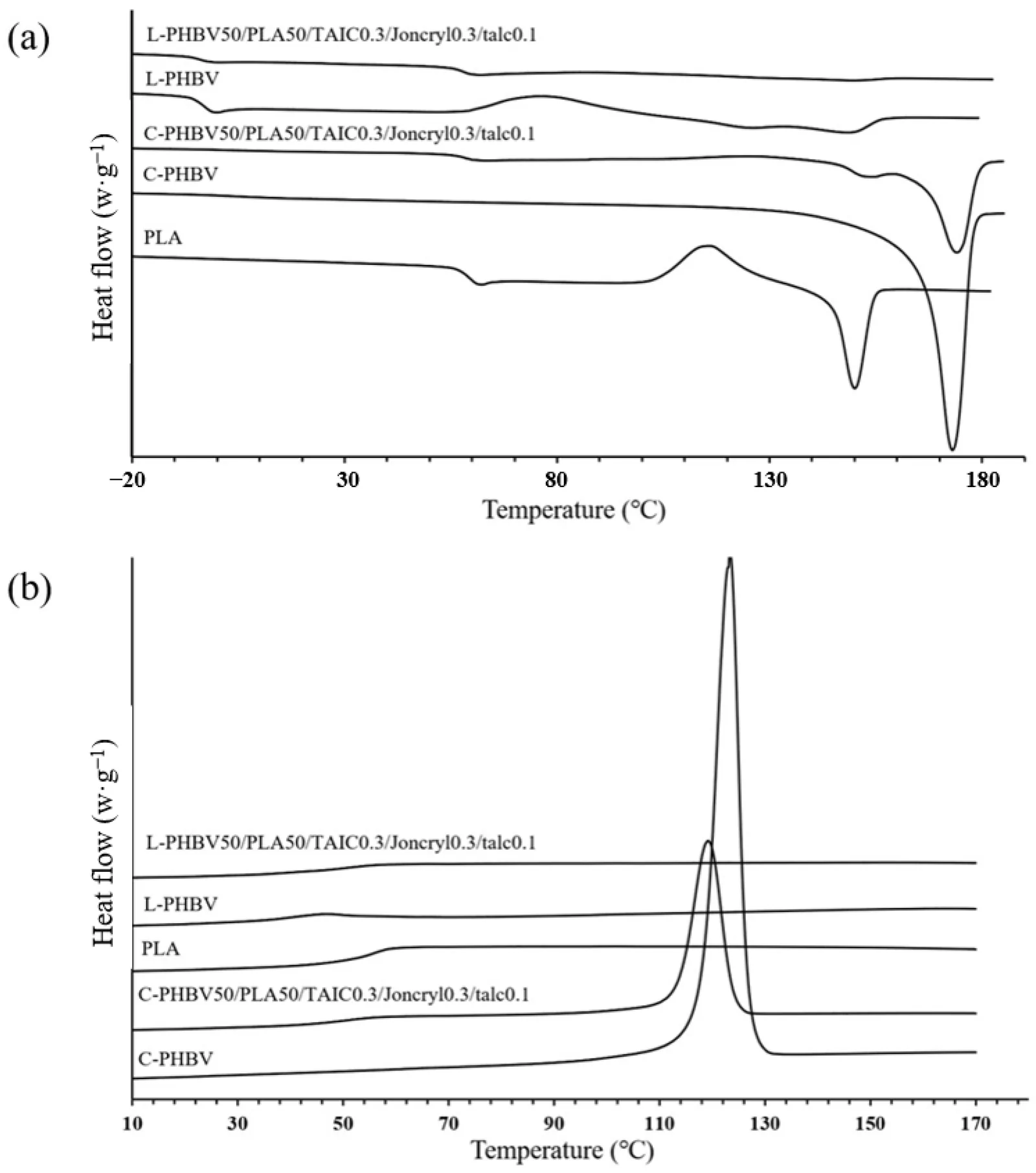
DSC thermograms of (**a**) the second heating cycle and (**b**) the second cooling cycle of the various injection-molded PHBV samples.

The neat and modified polymers were analyzed by DSC to determine their thermal properties including T_g_, T_m_, T_c_, and X_c_. The second heating and cooling DSC thermograms are shown in Figure 7. The T_g_, T_m_, and X_c_ values were obtained from the second heating cycle, while T_c_ was determined from the second cooling cycle (Table 3).

In contrast to L-PHBV and PLA, C-PHBV did not show a detectable T_g_. The T_g_ of the C-PHBV sample showed a slight reduction (1 °C) compared with neat PLA (58 °C vs. 60 °C). The L-PHBV-PLA blends showed two T_g_ values: the low-temperature T_g1_ (−4 °C), which was attributed to the L-PHBV phase, and a high-temperature T_g2_ (58.6 °C), which was attributed to the PLA phase. Both T_g_ values were slightly lower than those of the corresponding neat components. Specifically, the T_g1_ reduced from −3 °C in neat L-PHBV to −4 °C in the modified blend, while the T_g2_ reduced slightly from 60 °C in the neat PLA to 59 °C in the modified blend. These small shifts suggest changes in the local molecular environment and possible intermolecular interactions between L-PHBV and PLA. It is reported that the presence of two T_g_ values along with their slight reduction indicates partial miscibility between PLA with PHBV [93]. In fully immiscible systems, T_g_ values remain unchanged within the blend. The reduction in T_g_ of the PLA phase suggests that a fraction of L-PHBV chains entered in the amorphous PLA phase, increased free volume, and enhanced the chain mobility of the PLA. Also, low-molar mass and degraded L-PHBV chains act as plasticizers, further increasing chain mobility. As a result, the transition of PLA chains into the rubbery state occurs at a slightly lower temperature [93]. A similar plasticization effect occurred in the L-PHBV phase, where the degraded lower *M_w_* chains contribute to the reduction in T_g_ through in situ self-compatibilization mechanisms [93].

Unlike C-PHBV and PLA, neat L-PHBV showed two melting points: a low-temperature melting peak (T_m1_, 125 °C) and a high-temperature melting peak (T_m2_, 149 °C), which was attributed to isomorphism phenomena [11,34]. Due to the semicrystalline nature of PHBV, crystals with higher HV content contain a higher amorphous phase ratio and thus melt at lower temperature (T_m1_), whereas crystals with lower HV content contain higher crystallinity, and so melt at a higher temperature (T_m2_) [34,94]. Other factors contributing to two melting points include variations in crystalline morphology (e.g., thickness, perfection, lamellar stability, or distribution), crystallites of different molar masses, physical aging, and relaxation of the rigid amorphous phase [34,94].

In the L-PHBV blend, the lower melting peak disappeared, and a single melting peak was observed at 151 °C, which was slightly higher (1 °C) than that of neat L-PHBV. This is attributed to increased chain entanglement and branching induced by Joncryl and TAIC, which physically restrict chain mobility, along with possible partial miscibility between PLA and L-PHBV that hinders crystallization and chain alignment [30,95].

The C-PHBV showed two melting peaks at 152 °C and 174 °C, indicating the presence of two distinct polymer phases: a PLA-rich phase and a C-PHBV-rich phase. The lower melting peak (T_m1_) corresponds to the PLA-rich phase and was slightly higher (by 1.5 °C) than that of neat PLA, likely due to the nucleating effect of Talc and improved crystallization during processing [96]. The higher melting peak (T_m2_) corresponds to the C-PHBV-rich phase and remained almost similar to that of neat C-PHBV, suggesting that processing did not significantly alter its crystalline structure.

The X_c_ of the neat and modified samples were obtained from the first cooling cycle. Neat PLA was nearly amorphous (X_c_ = 0.2%). In the C-PHBV blend, the crystallinity of the C-PHBV-rich phase decreased significantly by 21% compared to the neat C-PHBV phase (40% vs. 64%), which can be attributed to branching and network formation induced by Joncryl and TAIC. Network formation reduces the amount of crystallizable material and restricts chain mobility, leading to decreased crystallinity [97]. In contrast, the X_c_ of the PLA-rich phase increased slightly (3.6% vs. 0.2%), which is consistent with the nucleating effect of talc [96]. Also, due to the higher melting temperature of C-PHBV than PLA, it can act as a nucleating agent for PLA, further promoting crystallization of the PLA phase [46].

In the L-PHBV blend, the crystallinity of L-PHBV dropped to nearly zero (0.08% vs. 2.8%), and PLA showed no detectable crystallinity. This behavior is attributed to increased chain entanglement and branching induced by Joncryl and TAIC, which physically restrict chain mobility, along with possible partial miscibility between PLA and L-PHBV, both of which hinder crystallization and chain alignment [30,95]. In addition, thermomechanical degradation of L-PHBV during processing, resulting in reduced molecular weight, also contributes to the decrease in crystallinity [98,99].

The I_C=O_ values of all neat and modified samples ranged from 2.13 to 3.64, which is consistent with previously reported values of 1.8 to 2.7 for PHBV (22%HV)- and of 2.2 to 3.3 for PHBV grafted with α-cellulose fibers [35]. Neat L-PHBV showed a lower I_C=O_ than neat C-PHBV (2.13 vs. 3.64), which can be attributed to its higher HV content, as higher HV content creates more amorphous, less crystalline material, resulting in a lower I_C=O_ index [100]. The I_C=O_ of the C-PHBV blend decreased significantly compared to neat C-PHBV (2.38 vs. 3.64), due to the branching and crosslinking effect of Joncryl and TAIC during reactive processing that disrupts crystallinity and increases the amorphous fraction, leading to the reduction in I_C=O_. In contrast, the L-PHBV blend showed a slight increase in I_C=O_ compared to neat L-PHBV (2.26 vs. 2.13), which is consistent with the reduction in its crystallinity to 0.08%, maintaining a predominantly amorphous structure, as observed in DSC results.

Neat PLA did not show a T_c_, while neat L-PHBV showed a T_c_ at 47 °C. However, the L-PHBV blend did not show a T_c_. The C-PHBV blend showed a slight reduction in T_c_ (3 °C) compared to neat C-PHBV (119 °C vs. 122 °C). The reduction in T_c_ of the C-PHBV blend can be attributed to kinetic and thermodynamic restrictions introduced by blending and the use of reactive additives. Generally, during high-shear melt blending, interactions between C-PHBV and PLA chains can restrict C-PHBV chain mobility and limit the ability of chains to reorganize into ordered crystalline structures, thus leading to reduced overall crystallinity and a lower T_c_ [99,101,102]. In addition, TAIC and Joncryl disrupt chain regularity through chain extension and network formation, introducing steric hindrance and further restricting molecular mobility. This limits the parallel chain alignment required for crystallization, increasing the degree of undercooling needed to overcome the kinetic barrier, leading to a reduced T_c_ [30,95]. Although talc acts as a nucleating agent, its effect is outweighed by the combined influence of blending, high-shear processing, and network formation within the blend.

**Figure 8:**
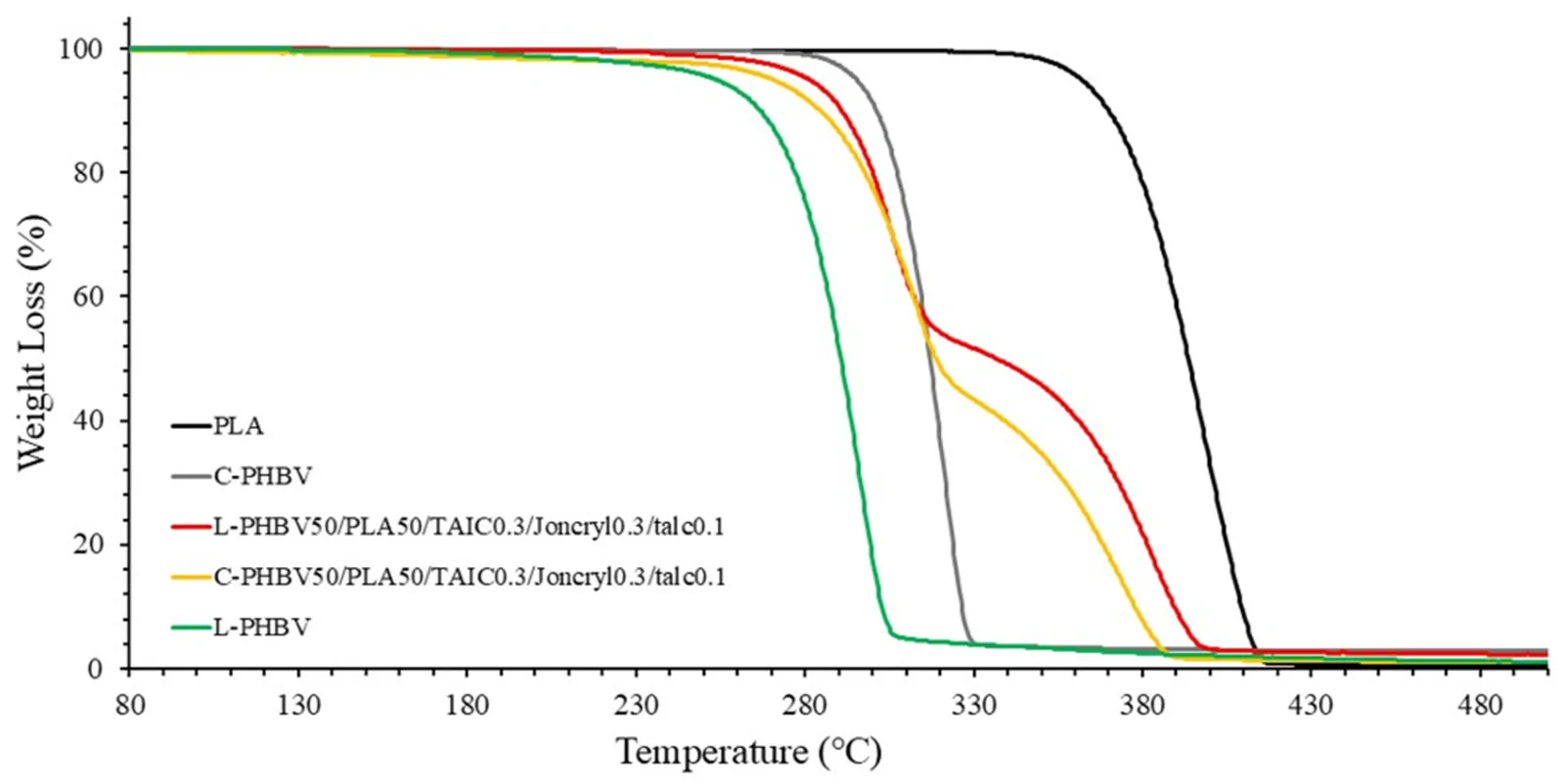
TGA thermograms for various injection-molded PHBVs.

TGA analysis was conducted to evaluate the thermal degradation behavior of the neat and modified PHBV samples (Table 3, and Figure 8). Based on the derivative thermogravimetric (DTG) curves, the onset temperature of significant mass loss (T_onset_), the endset temperature at which the mass loss is essentially complete (T_endset_), and the temperature corresponding to the occurrence of maximum weight loss (T_d_) were determined. T_onset_ reflects the initial thermal stability of the polymer, whereas T_endset_ indicates the completion of the main degradation process. All neat polymers exhibited a single degradation T_d_, located at 322 °C for C-PHBV, 299 °C for L-PHBV, and 402 °C for PLA, indicating a one-step degradation process for each polymer. In contrast, the L-PHBV and C-PHBV samples showed a two-step degradation behavior. The first degradation step (T_d1_) is associated with PHBV degradation, while the second step (T_d2_) is associated with PLA degradation. This indicates that PHBV and PLA form an immiscible or partially miscible blend, which is typical behavior for the blend of these two polymers [103,104]. In the C-PHBV blend sample, both the T_d1_ (309 °C) and the T_d2_ (374 °C) were lower than the corresponding values for neat C-PHBV and neat PLA by 13 °C and 28 °C, respectively. The reduction in T_onset_ and T_d_ can be attributed to chain scission reactions occurring during reactive melt processing, including thermomechanical degradation, which take place alongside network formation. These processes result in an increased PDI value in the C-PHBV blend compared to the neat polymers. A higher PDI value in the C-PHBV blend indicates a higher fraction of low molecular weight chains, which requires less energy to degrade, thereby reducing T_onset_ and Td [105,106]. A lower T_onset_ in crosslinked systems compared to their constituent polymers is also reported in previous studies [105]. In the L-PHBV blend, the degradation temperature of the L-PHBV fraction (T_d1_) was higher relative to neat PHBV (306 vs. 299 °C). In contrast, the degradation temperature of the PLA fraction (T_d2_) reduced compared to neat PLA (382 vs. 402 °C). The reduced separation between the degradation steps of L-PHBV and PLA can be attributed to partial compatibility between the two polymers, with PLA providing a barrier effect that slows the thermal degradation of the L-PHBV fraction [103]. Also, both T_d1_ and T_d2_ in the L-PHBV blend were higher than those in the C-PHBV blend, which can be attributed to the higher Mw and lower PDI value of the L-PHBV blend [106]. Comparison of T_onset_ values indicates that neat PLA is inherently more thermally stable than neat PHBVs. The T_onset_ for neat PLA (365 °C) is significantly higher than that of neat C-PHBV (292 °C) and L-PHBV (267 °C). Similarly, neat PLA showed a higher T_endset_ (415 °C) compared to neat C-PHBV (329 °C) and L-PHBV (307 °C). PHBV typically undergoes single-step thermal degradation via a nonradical random chain scission mechanism (β-elimination) involving a six-membered ring transition state [34,50,107,108,109]. This process produces crotonic acid and oligomers with crotonyl end groups, which may further degrade to propylene, CO_2_, acetaldehyde, and ketene [110]. The T_d1_ values reported in Table 3 are consistent with this mechanism. In contrast, PLA exhibits a more complex degradation behavior involving multiple overlapping pathways as the temperature increases [111]. Its thermal stability is influenced by variables such as temperature, molecular weight, mechanical stress, oxygen, moisture, hydrolysis products, and residual materials [112,113]. During melt processing, these variables collectively contribute to thermomechanical, thermal, and hydrolytic degradation [113]. Generally, the degradation products of PLA include lactide, acetaldehyde, carbon monoxide, and CO_2_ [111].

In polymer blends, the difference between the highest T_m_ (by DSC) of the components, to ensure complete melting and mixing, and the lowest T_onset_ (by TGA) provides the processing window for molding and reactive melt processing of polymers [114]. Within this temperature range, significant degradation is not expected [115].

The processing window in the C-PHBV blend was slightly lower than that of the neat C-PHBV (115 °C vs. 119 °C). In contrast, the L-PHBV blend showed an increase of 16 °C in the processing window. This difference can be due to the simultaneous effect of network formation and chain scission reactions, as well as the lower *M_w_* and higher PDI value of the C-PHBV blend compared to L-PHBV [105,106]. Additionally, the difference between T_onset_ and T_endset_ increased by 60 °C and 70 °C for the C-PHBV and L-PHBV blends, respectively, compared to their neat counterparts. This suggests that degradation in the optimized blends occurs over a broader temperature range and at a slower rate than in the neat polymers.

### 3.5. Mechanical Properties

Tensile properties provide insight into how materials respond under load. Table 4 shows the tensile properties of the neat PHBVs, neat PLA, and the corresponding modified PHBV blends (Supplementary Materials Figure S2). As shown in Table 4, neat PLA and neat C-PHBV showed significantly higher tensile strengths (39.7 MPa and 32.8 MPa, respectively) than neat L-PHBV (13.2 MPa). The lower tensile strength of neat L-PHBV compared with neat C-PHBV can be attributed to its considerably higher HV content (28% vs. 8%). Higher HV content is generally associated with reduced crystallinity, tensile strength, stiffness, and increased flexibility [10,34,40,116]. The first DSC heating scan, which indicates thermal history of yet-processed samples, showed X_c_% of 3.9% for neat L-PHBV, and 60.1% for C-PHBV, supporting the contribution of crystalline structure to the higher tensile strength of C-PHBV.

The addition of PLA and reactive modification produced different effects depending on the PHBV source. The tensile strength of the C-PHBV blend (28.9 MPa) was lower than neat C-PHBV (32.8 MPa). This reduction is consistent with the reduction in the crystallinity of the C-PHBV phase from 60.1% in neat C-PHBV to 53.5% in the C-PHBV blend, based on the first DSC heating scan. Therefore, the reduction in crystallinity may have contributed to the reduction in tensile strength. Previous studies have shown that the addition of PLA can affect the mechanical properties of PHBV-PLA blends, with the resulting properties depending on blend composition and characteristics [38,104].

In addition to crystallinity, molecular weight changes provide important insight into the mechanical response of the C-PHBV formulation. The approximately 50% reduction in *M_w_* from neat C-PHBV to the C-PHBV blend, together with the increased PDI value from 1.1 to 1.47, indicates a substantial molecular weight degradation, showing thermomechanical and shear-induced chain scission during melt processing. The PDI describes the breadth of molar mass distribution of polymers, and also plays a critical role in polymer mechanical properties. An increase in the PDI value, particularly when accompanied by a reduction in *M_w_*, indicates a broader distribution containing a greater fraction of lower molecular weight chains. These shorter chains can act as plasticizers, reduce entanglement density and intermolecular interactions, and increase chain mobility and free volume within the polymer matrix, potentially contributing to reductions in both tensile strength and stiffness [40,117,118]. Although the incorporation of TAIC/Joncryl into the C-PHBV blend induced a limited network formation (3.73% gel fraction) and evidence of chemical modification via FTIR, the extent of this network was insufficient to offset the loss of mechanical properties driven by degradation reactions followed by molecular weight reduction.

The L-PHBV blend showed a different mechanical response. The tensile strength of the L-PHBV blend (24.3 MPa) was higher than that of neat L-PHBV (13.2 MPa). This is consistent with the substantial increase in crystallinity from 3.9% in neat L-PHBV to 21.0% in the blend based on the first DSC heating scan. The development of a more crystalline structure during processing may therefore have contributed to improved tensile strength. The combination of increased tensile strength in the L-PHBV blend indicates a shift toward a stronger material compared with neat L-PHBV. Considering the lack of measurable insoluble fraction in the L-PHBV blend, the improved tensile strength may be attributed to the reinforcing effect of PLA within the blend.

Comparison of the two reactive modified blends further demonstrates the influence of crystallinity on the resulting mechanical properties. The C-PHBV blend showed slightly higher tensile strength (28.9 MPa) than the L-PHBV blend (24.3 MPa). The C-PHBV blend also showed substantially higher crystallinity of the PHBV phase (53.5%) than the L-PHBV blend (21.0%) in the first DSC heating scan. The higher crystalline fraction may have partially contributed to the relatively higher stiffness of the C-PHBV blend. Other parameters, such as PHBV’s molecular characteristics and its different responses to reactive processing, could have contributed to the differences observed in mechanical behavior.

## 4. Conclusions

In this work, PHBV was successfully produced from fermented dairy manure using mixed microbial culture, and then extracted and purified. Next, the purified L-PHBV was blended with PLA, TAIC, Joncryl, and talc via reactive melt processing to enhance its processability and overall performance for packaging applications. The results were compared with a C-PHBV blend processed under similar reactive melt processing conditions. The addition of TAIC, Joncryl, and talc significantly affected the structure–property relationships of both PHBV blends. The optimized composition containing 0.3 wt% TAIC, 0.3 wt% Joncryl, and 0.1 wt% talc showed the highest complex viscosity among the evaluated PHBV/PLA formulations, as confirmed by dynamic rheological measurements, suggesting improved melt processability. Although thermomechanical degradation during processing overcame the formation of an insoluble network leading to reduction in the molar mass of all samples, with a more significant reduction in the L-PHBV, the modified blends showed a significant increase in complex viscosity due to enhanced intermolecular interactions. Different modification mechanisms were identified for the two blends. In the C-PHBV/PLA blend, FTIR spectral and gel fraction analyses provided evidence consistent with the formation of a limited insoluble fraction, which is potentially associated with chemical reactions involving TAIC and Joncryl. In contrast, the L-PHBV/PLA blend showed no measurable gel formation and its enhanced rheological performance was mainly attributed to increased chain entanglement, higher HV content, and possible partial miscibility with PLA.

Thermal analysis indicated a reduction in crystallinity in both modified C/L-PHBV/PLA blends, with L-PHBV showing a nearly amorphous structure and an increased processing window. Mechanical results showed a significant increase in tensile strength in the modified L-PHBV blend, whereas the modified commercial PHBV blend showed a slight reduction in tensile strength attributed to chain scission during reactive processing. Considering the improved properties, the composition of PHBV50/PLA50/TAIC0.3/Joncryl 0.3/TALC0.1 for C/L-PHBV could be suitable for blown-film manufacturing and a sustainable alternative for packaging applications.

## Figures and Tables

**Table 1:** Power law parameters (*K* and *n*) and complex viscosity (η*) at ω = 6.28 rad·s^−1^ (1 Hz) determined at 180 °C for the neat PHBVs, neat PLA, and optimized PHBV50-PLA50 compositions.

Sample	*K* (Pa·S)	*n*	R^2^	η* (Pa·s)
C-PHBV	16.3	0.800	0.46	11.3
PLA	795	0.903	0.92	665
L-PHBV	1.55	0.448	0.65	0.56
C-PHBV50/50PLA/0.3 TAIC/0.3 Joncryl/0.1talc	338	0.824	0.98	273
L-PHBV50/50PLA/0.3 TAIC/0.3 Joncryl/0.1talc	299	0.791	0.92	203

**Table 2:** Molar mass and polydispersity index values of neat PHBVs, PLA, and optimum modified compositions.

Sample	Molar Mass ^1^		
	*M_w_*	*M_n_*	PDI
	(×10^5^ g·mol^−1^)	(×10^5^ g·mol^−1^)	
PLA-pellet	1.5 ± 0.1	1.3 ± 0.0	1.14 ± 0.00
C-PHBV-pellet	3.1 ± 0.0	2.6 ± 0.0	1.17 ± 0.00
L-PHBV-pellet	8.2 ± 0.3	7.0 ± 0.2	1.44 ± 0.38
PLA molded	1.8 ± 0.0	1.6 ± 0.0	1.20 ± 0.00
C-PHBV molded	2.9 ± 0.0	2.6 ± 0.0	1.10 ± 0.00
L-PHBV molded	3.1 ± 0.1	2.6 ± 0.1	1.20 ± 0.00
L-PHBV 50/PLA 50	3.8 ± 0.6	3.2 ± 0.5	1.18 ± 0.00
C-PHBV 50/PLA 50	1.8 ± 0.2	1.6 ± 0.1	1.14 ± 0.06
C-PHBV 50-0.3 TAIC-0.3 Joncryl-0.1talc	1.4 ± 0.0	1.0 ± 0.0	1.47 ± 0.06
L-PHBV 50-0.3 TAIC-0.3 Joncryl-0.1talc	3.4 ± 0.1	2.7 ± 0.1	1.20 ± 0.00

^1^ Values are mean standard deviation.

**Table 3:** Physical and thermal properties of neat PHBV, neat PLA, and PHBV50/PLA50 compositions.

Sample			DSC							FTIR	TGA			
	T_g1-PHBV_	T_g2-PLA_	T_m1_	T_m2_	T_c_	X_cPLA_^1^	X_cPHBV_^1^	X_cPLA_	X_cPHBV_	I_C=O_	T_onset_	T_d1-PHBV_	T_d2-PLA_	T_endset_
	(°C)	(°C)	(°C)	(°C)	(°C)	(%)	(%)	(%)	(%)		(°C)	(°C)	(°C)	(°C)
PLA	-	59.7 ± 0.4	150.2 ± 0.2	-	-	0.47	-	0.2	-	-	365 ± 2.0	-	402 ± 2.0	415 ± 2
C-PHBV	-	-	-	173.2 ± 0.2	122.5 ± 1.1	-	60.1	-	64.3	3.64	292 ± 1	322 ± 1	-	329 ± 0.0
L-PHBV	−3.1 ± 0.2	-	125.2 ± 1.4	149.5 ± 0.3	46.6 ± 0.1	-	3.9	-	2.8	2.13	267 ± 2	299 ± 4	-	307 ± 4
C-PHBV50/PLA50/0.3 TAIC/0.3Joncryl/0.1talc	-	58.3 ± 0.1	151.7 ± 0.3	173.7 ± 0.6	119.4 ± 0.3	-	53.5	3.6	40.1	2.38	289 ± 5	309 ± 1	374 ± 0.0	386 ± 2
L-PHBV50/PLA50/0.3 TAIC/0.3Joncryl/0.1talc	−4.0 ± 0.1	58.6 ± 1.0	150.7 ± 1.1	-	-	-	21.0	-	0.08	2.26	284 ± 0	306 ± 2	382 ± 1.0	394 ± 2

^1^ Crystallinity calculated from the first DSC heating scan.

**Table 4:** Tensile properties of neat PHBVs, neat PLA, and optimized PHBV blend samples ^1,2^.

Sample	Tensile Strength
	(MPa)
PLA	39.7 ± 13.4 ^a^
C-PHBV	32.8 ± 1.3 ^ab^
L-PHBV	13.2 ± 0.5 ^c^
C-PHBV50/PLA50/0.3 TAIC/0.3Joncryl/0.1talc	28.9 ± 5.5 ^b^
L-PHBV50/PLA50/0.3 TAIC/0.3Joncryl/0.1talc	24.3 ± 4.9 ^b^

^1^ Values are mean ± standard deviation. ^2^ Statistical differences of means using a Tukey test (*p*-value < 0.05); different superscript letters (a, b and c) indicate significance.

## Data Availability

The original contributions presented in this study are included in the article. Further inquiries can be directed to the corresponding author.

## Supplementary Materials

The following supporting information can be downloaded at: https://www.mdpi.com/article/10.3390/ma19204406/s1. Figure S1. Complex viscosity (η*) versus shear rate (ω) plots for all of compositions made with neat PHBVs, neat PLA, and PHBV50-PLA50 blends (180 °C). Figure S2. Representative tensile stress-strain curves for PLA, PHBV-C, PHBV-L, PHBV-C50/50PLA/0.3 TAIC/0.3/Joncryl/0.1talc and PHBV-L-50/50PLA/0.3 TAIC/0.3 Joncryl/0.1talc samples.
