# Supplementary material for: Modification of Poly(3-hydroxybutyrate-co-3-hydroxyvalerate)/Poly(lactic acid) Blends by In-Situ Reactive Melt Processing for Sustainable Packaging Application
Source: Materials (Basel). 2026 Oct 20;19(20):4406. doi: 10.3390/ma19204406 (PMC13706792; doi:10.3390/ma19204406)
Supplement: Supplementary file 1 [file materials-19-04406-s001.zip › materials-4557122-supplementary.pdf]

Modification of Poly(3-hydroxybutyrate-co-3-hydroxyvalerate)/Poly(lactic acid) Blends by In-Situ Reactive Melt Processing for Sustainable Packaging Application

Maryam Abbasi[1], Erik R. Coats[2] and Armando G. McDonald[1,*]

[1] Department of Forest, Rangeland and Fire Sciences, University of Idaho, Moscow, ID 83844, USA
[2] Department of Civil and Environmental Engineering, University of Idaho, Moscow, ID 83844, USA; ecoats@uidaho.edu
[*] Correspondence: armandm@uidaho.edu; Tel.: +1-208-885-9454

Supplemental Material

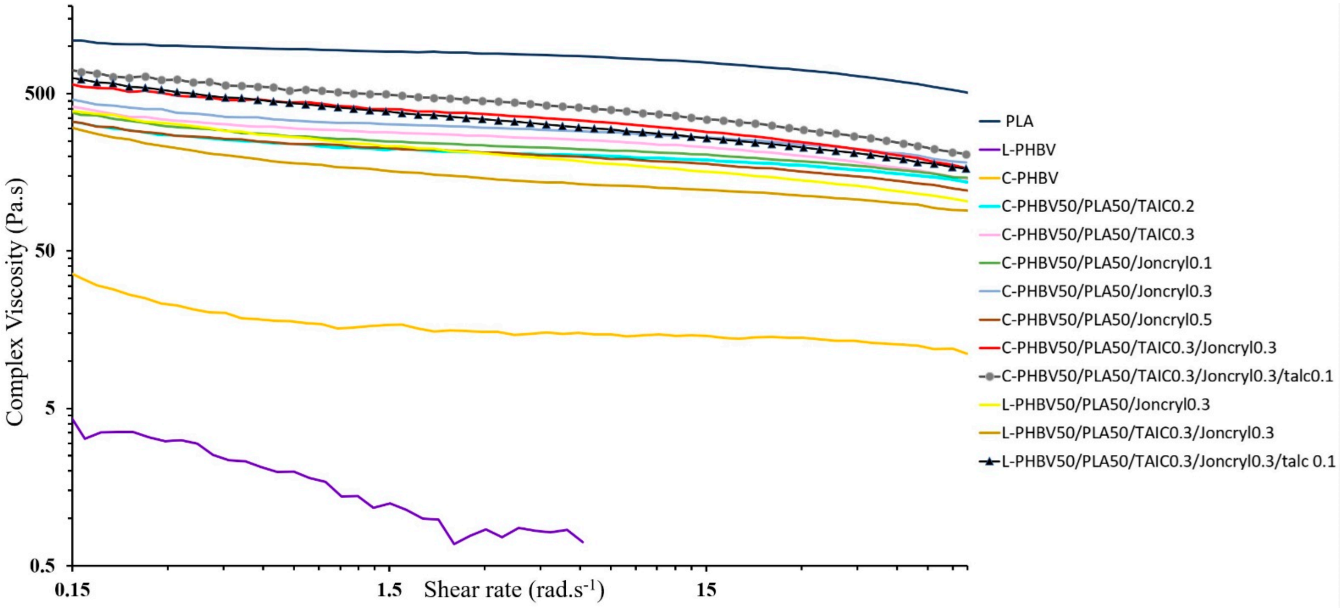

Figure S1. Complex viscosity (η*) versus shear rate (ω) plots for all of compositions made with neat PHBVs, neat PLA, and PHBV50-PLA50 blends (180 °C).

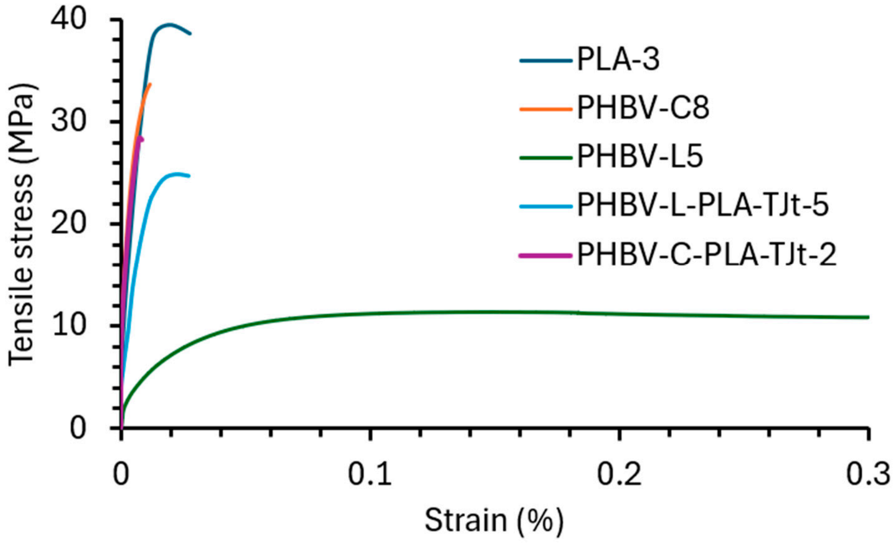

Figure S2. Representative tensile stress-strain curves for PLA, PHBV-C, PHBV-L, PHBV-C50/50PLA/0.3 TAIC/0.3/Joncryl/0.1talc and PHBV-L-50/50PLA/0.3 TAIC/0.3 Joncryl/0.1talc samples.
